# Aspartate β-hydroxylase expression promotes a malignant pancreatic cellular phenotype

**DOI:** 10.18632/oncotarget.2840

**Published:** 2014-11-26

**Authors:** Xiaoqun Dong, Qiushi Lin, Arihiro Aihara, Yu Li, Chiung-Kuei Huang, Waihong Chung, Qi Tang, Xuesong Chen, Rolf Carlson, Christina Nadolny, Gregory Gabriel, Mark Olsen, Jack R. Wands

**Affiliations:** ^1^ Department of Biomedical and Pharmaceutical Sciences, College of Pharmacy, The University of Rhode Island, Kingston, RI, USA; ^2^ Current address: Department of Internal Medicine, College of Medicine, The University of Oklahoma Health Sciences Center, Oklahoma City, OK, USA; ^3^ Liver Research Center, Rhode Island Hospital, Warren Alpert Medical School, Brown University, Providence, RI, USA; ^4^ Department of Chemistry and Biochemistry, Kennesaw State University, Kennesaw, GA, USA; ^5^ Department of Pharmaceutical Sciences, College of Pharmacy–Glendale, Midwestern University, Glendale, Arizona, USA

**Keywords:** Aspartate β-Hydroxylase, oncogenesis, small molecule inhibitor (SMI), malignant phenotype, pancreatic cancer

## Abstract

Pancreatic cancer (PC) is one of the leading causes of cancer related deaths due to aggressive progression and metastatic spread. Aspartate β-hydroxylase (ASPH), a cell surface protein that catalyzes the hydroxylation of epidermal growth factor (EGF)-like repeats in Notch receptors and ligands, is highly overexpressed in PC. ASPH upregulation confers a malignant phenotype characterized by enhanced cell proliferation, migration, invasion and colony formation *in vitro* as well as PC tumor growth *in vivo*. The transforming properties of ASPH depend on enzymatic activity. ASPH links PC growth factor signaling cascades to Notch activation. A small molecule inhibitor of β-hydroxylase activity was developed and found to reduce PC growth by downregulating the Notch signaling pathway. These findings demonstrate the critical involvement of ASPH in PC growth and progression, provide new insight into the molecular mechanisms leading to tumor development and growth and have important therapeutic implications.

## INTRODUCTION

Pancreatic cancer (PC) is the 4^th^ leading cause of cancer-related death in USA, with an estimated 45,220 new cases and 38,460 deaths in 2013 [1, 2]. Despite improvements in detection and management, the 5-year survival rate of PC is only 5-6%. At diagnosis, 80-85% of patients present with advanced unresectable disease due to invasion and widespread metastasis. Pancreatic cancer is notoriously aggressive and refractory to treatment [1, 2]. There is a need to explore the molecular mechanisms of PC development, progression and metastasis in an attempt to identify new molecular targets for therapy. Aspartate β-hydroxylase (ASPH) is an ∼86KD Type II transmembrane protein and a member of the α-ketoglutarate-dependent dioxygenase family [3, 4]. ASPH catalyzes the β-hydroxylation of aspartyl and asparaginyl residues in epidermal growth factor (EGF)-like repeats of various proteins such as Notch, Jagged (JAG) and Delta-like (DLL) [5]. Notch signaling plays critical roles in cell growth, differentiation, adhesion, migration and metastasis [6].

This study examines the expression of ASPH in human PC cell lines and pancreatic ductal adenocarcinoma (PDAC) tumor tissues, as well as determines how ASPH activates Notch signaling as a major contributor to generation of malignant phenotypes. We demonstrated that ASPH exerts effector function by promoting cell proliferation, migration, invasion, and malignant transformation. More important, PC tumor growth and progression may be altered by a small molecule inhibitor (SMI) of ASPH β-hydroxylase activity.

## RESULTS

### Overexpression of ASPH generates a malignant phenotype

**Fig. 1:**
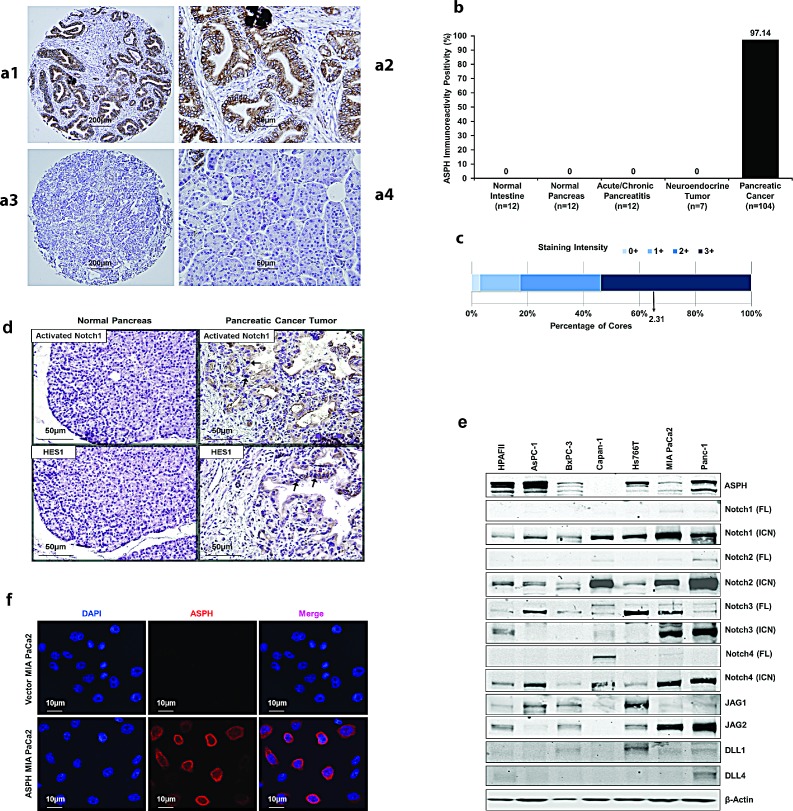
ASPH expression in human PDAC, pancreatic neuroendocrine tumor, acute and chronic pancreatitis, normal pancreas and normal intestine (a1) Typical ASPH expression in PDAC at 40× in tissue microarrays (TMAs) from 104 tumors. (a2) A representative example of IHS with mAb FB-50 (400×). (a3) A representative PDAC at 40× stained with a negative control mAb against hepatitis B surface antigen of the same isotype as mAb FB-50. (a4) Normal adjacent pancreas stained with the anti-ASPH FB-50 mAb (400×). (b) A summary of positive and negative expression of ASPH by IHS in normal tissues, pancreatitis and pancreatic tumors. (c) A bar graph of staining intensity with a mean score of 2.31 for PDACs where 0 = no, 1 = weak, 2 = moderate, 3 = strong expression of ASPH by IHS. Fig. 1. (d) Activated Notch1 and HES1 were also overexpressed in the cytoplasm and nuclei of tumor tissue compared to adjacent normal pancreas of PC patients (400×). The arrows highlight the positive staining of activated Notch1 and HES1 in the nuclei of tumor cells in representative examples. (e) Expression profiling of ASPH and Notch signaling proteins in 7 human PC cell lines. Most PC cell lines showed upregulation and activation of C-terminal intracellular Notch (ICN) 1, 2, 4 and to a lesser extent, Notch 3 as determined by Western blot analysis. The Notch ligands Jagged (JAG) 1 and 2 were expressed in 4/7 and 5/7 of the cell lines, respectively. In contrast, Delta (DLL) 1 and 4 were infrequently upregulated. (f) Lentiviral transfection and stable expression of ASPH in MIA PaCa2 cells. Note the cell surface localization of ASPH after stable overexpression by lentivirus as depicted in the middle panel as compared to the empty vector stable transfected control.

The level of ASPH expression was determined by immunohistochemical staining (IHS) in human pancreatic ductular adenocarcinomas (PDACs) (n=104), pancreatic neuroendocrine tumors (n=7), as well as acute or chronic pancreatitis (n=12), compared to adjacent normal pancreas (n=12), and normal intestine (n=12) as shown in Fig. 1. There was substantial ASPH protein expression in 101 of 104 PDACs (97.1%) on tissue microarrays (TMAs), and no immunoreactivity was observed in pancreatitis, normal pancreas, intestine or pancreatic neuroendocrine tumors (Fig. 1a, b). Most PDACs had a strong staining intensity (Fig. 1c). Activated Notch1 and HES1 were also overexpressed in the cytoplasm and nuclei of PDAC tumor cells (Fig. 1d).

The expression profiling of ASPH in 7 human PC cell lines was examined by Western blot analysis. High levels of the 86kD ASPH proteins were found in HPAFII, AsPC-1, Hs766T and Panc-1; very low levels were observed in BxPC-3 and MIA PaCa2 and little, if any, expression was noted in Capan-1 cells. Expression profiling of Notch signaling proteins is presented for comparison in Fig. 1e. We prepared lentiviral mediated stable transfection of ASPH that localized to the cell surface in MIA PaCa2 cells (Fig. 1f) with very low endogenous levels of ASPH as described [7, 8]. Empty vector transfected cells served as the control.

**Fig. 2:**
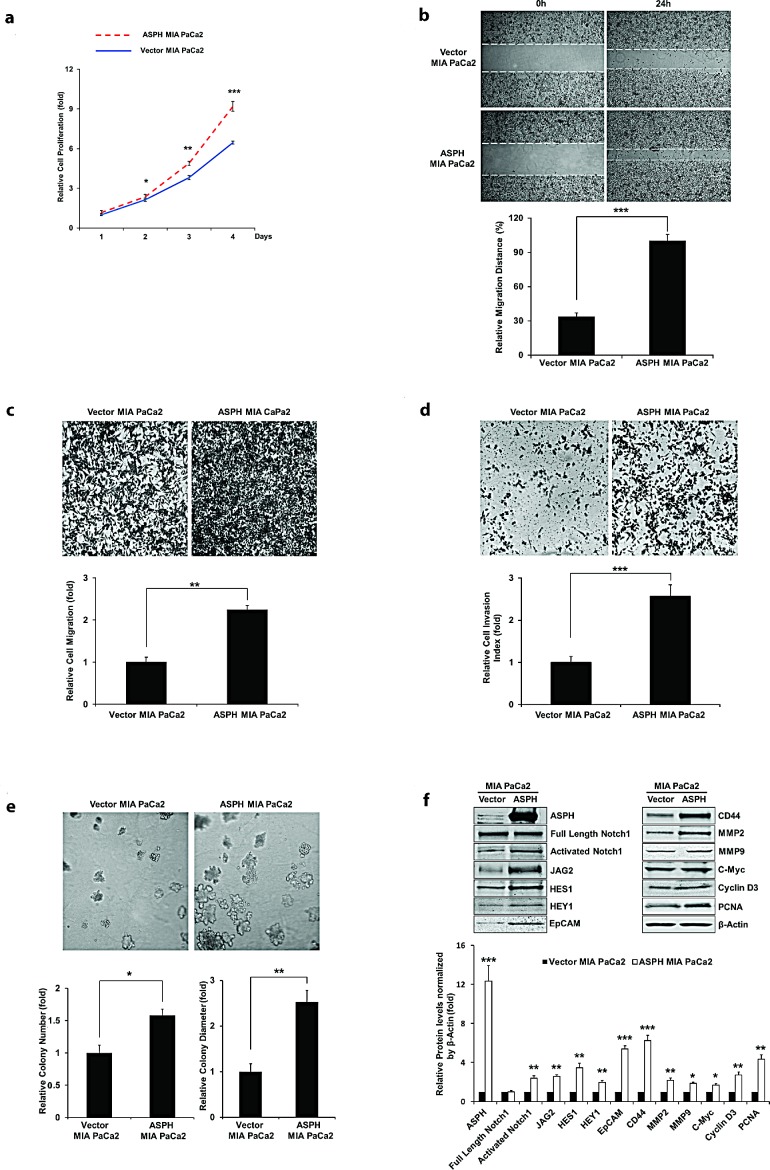
Stable overexpression of ASPH in MIA PaCa2 cells promotes cell proliferation (a), wound healing (b), migration (c), invasion (d), and colony formation (e) as indices of changes in the malignant phenotype. ASPH overexpression also enhances the expression of activated Notch1, JAG2, and Notch signaling downstream target genes including HES1, HEY1, EpCAM, CD44, c-Myc, MMP2/9, cyclin D3 and PCNA as shown in Western blot analysis (f). ^*^p<0.05; ^**^p<0.01; ^***^p<0.001.

**Fig. 3:**
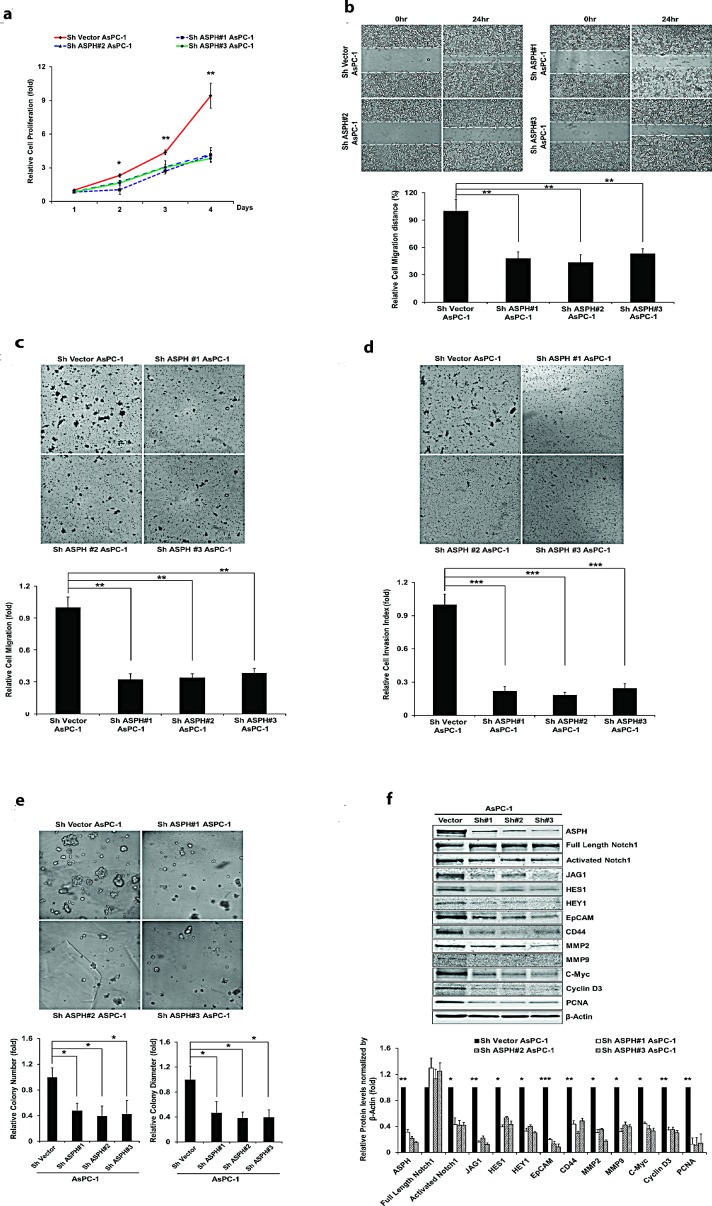
Knockdown of ASPH by specific shRNAs in AsPC-1 cells inhibited (a) proliferation, (b) wound healing, (c) migration, (d) invasion, and (e) colony formation as indices of changes in the malignant phenotype. (f) Endogenous overexpression of ASPH was associated with upregulation of activated Notch1 and JAG2, as well as Notch signaling downstream target genes HES1, HEY1, EpCAM, CD44, c-Myc, MMP2/9, cyclin D3 and PCNA in a lentiviral transfected stable cell line expressing a nonspecific scrambled shRNA. The AsPC-1 cell lines stable transfected with ASPH specific shRNAs demonstrated downregulation of activated Notch1, JAG2, and Notch activated genes as determined by Western blot analysis. *p<0.05; **p<0.01; ***p<0.001.

Figure 2 panel a-e demonstrate the biological effects of ASPH overexpression on the PC cell phenotype on MIA PaCa2 as measured by proliferation, migration, invasion, as well as colony formation as an index of malignant transformation. Similar findings were evident with stable ASPH transfected Capan-1 cells (data not shown). This aggressive malignant phenotype was significantly attenuated by specific shRNAs “knockdown” of ASPH in AsPC-1 cells with high endogenous levels as demonstrated by a reduction in proliferation, migration, invasion and colony formation (Fig. 3, panel a-e).

### β-hydroxylase activity is necessary for ASPH to function as a transforming protein

The C-terminus of ASPH contains the amino acid (aa) sequence of the catalytic site, (M^670^HPGTH^675^) and its sequence is identical in human, rat, mouse and cattle [9]. The H^675^ aa is specifically involved in Fe^2+^ coordination and is critical for its enzymatic activity; H^675^ aa is also highly conserved in the chicken and fly [10].

**Fig. 4:**
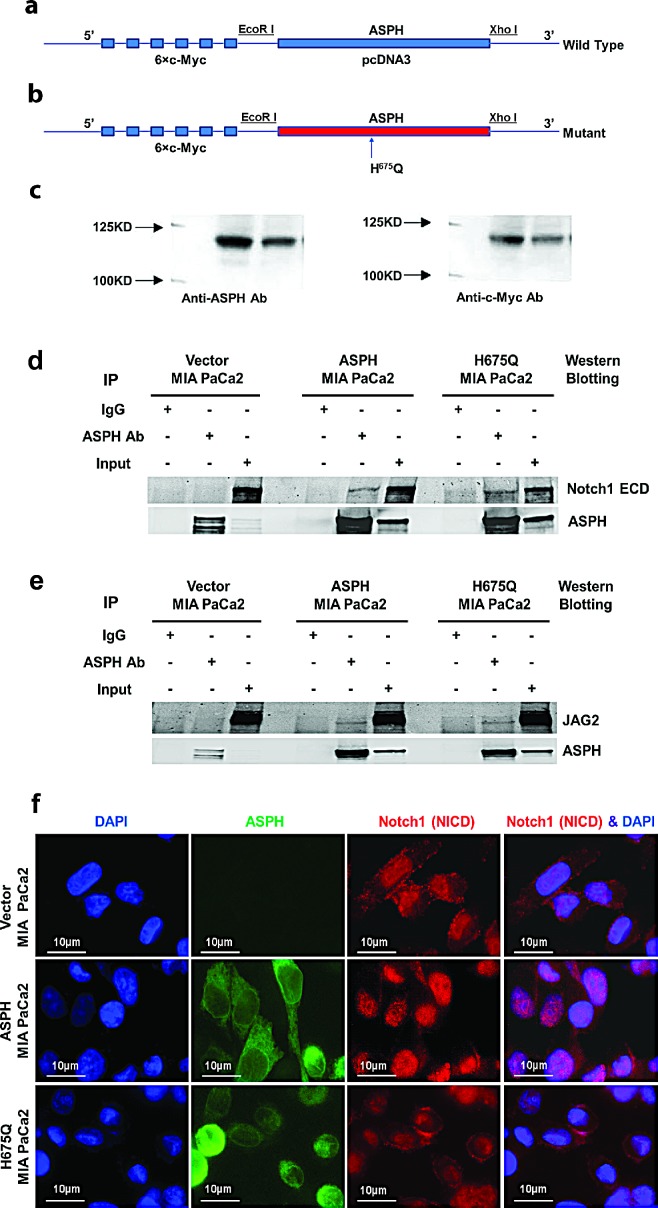
ASPH gene was subcloned into pcDNA3 with 5′ c-Myc tagged vector. Inclusion of a c-Myc-tag allowed discrimination of exogenous expressed protein from endogenous cellular levels (a) “Wild type” (WT) ASPH construct; (b) ASPH mutant (H^675^Q) construct where enzymatic activity was reduced to 20% of “wild type” protein level; (c) Western blot analysis of WT and mutant proteins with antibodies to ASPH and c-Myc. A direct physical interaction of ASPH with (d) Notch1 or (e) JAG2 by co-immunoprecipitation (IP) was observed in MIA PaCa2 cells. ASPH, Notch1, and JAG2 immunoprecipitates were fractionated by SDS-PAGE and analyzed by Western blot using the FB-50 monoclonal antibody. The negative control (Neg) corresponds to an IP result using non-relevant antibody to hepatitis B virus. (f) Microscopy showing increased nuclear translocation of activated Notch1 ICN in the nuclei of MIA PaCa2 cells stably expressing WT-ASPH. Note that the mutant H^675^Q ASPH substantially reduces nuclear localization of Notch1 ICN comparable to that in vector transfected MIA PaCa2 cells, which suggests that the enzymatic activity of the protein is critical for Notch activation.

**Fig. 5:**
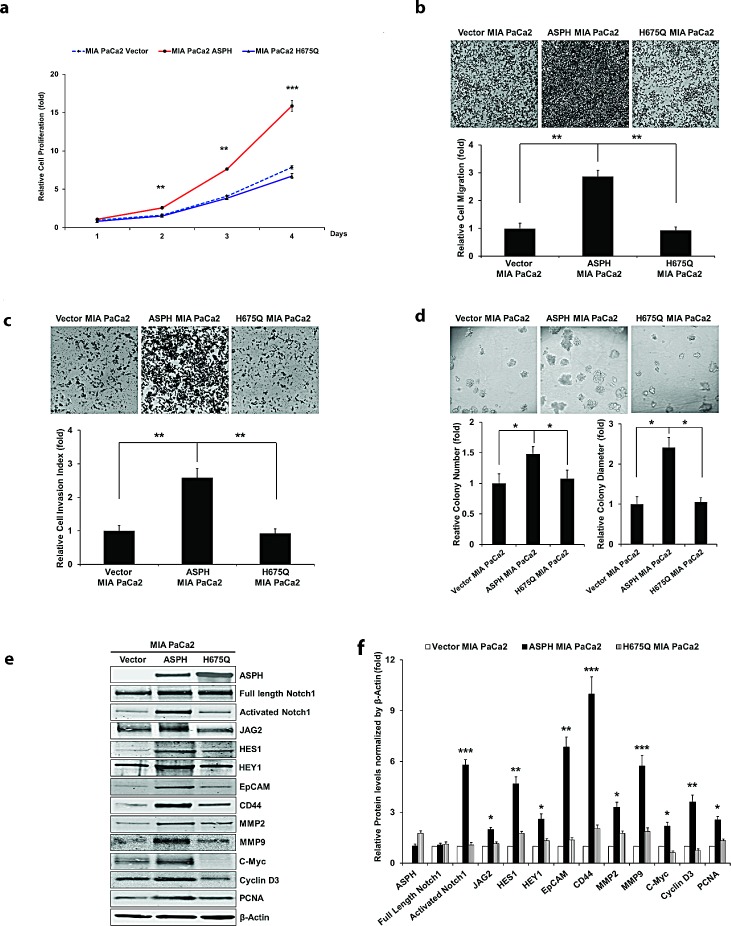
The β-hydroxylase activity of ASPH is required for its transforming activity MIA PaCa2 cells were stably transfected with empty vector (EV), “wild type” (WT)-ASPH, or H^675^Q-ASPH mutant and the levels of expression were confirmed by Western blot. There is low-level endogenous ASPH in MIA PaCa2 cells as shown here and in Fig. 1. Effects of EV, WT-ASPH and H^675^Q-ASPH on (a) cell proliferation, (b) migration, (c) invasion, and (d) colony formation are significantly different. (e and f) represents a Western blot demonstrating WT-ASPH induced activation of Notch signaling as determined by increased levels of Notch1 ICN, JAG2, as well as increased expression of downstream responsive genes HES1, HEY1, EpCAM, CD44, c-Myc, MMP2/9, cyclin D3 and PCNA. In contrast, the mutant H^675^Q ASPH construct shows significantly reduced activated Notch1 ICN, JAG2, as well as HES1, HEY1, EpCAM, CD44, c-Myc, MMP2/9, cyclin D3 and PCNA. The results suggest that the β-hydroxylase activity of ASPH is essential for Notch signaling activation. ^*^p<0.05; ^**^p<0.01; ^***^p<0.001.

We have established WT-ASPH and H^675^Q mutant expression constructs (Fig. 4a, b, c). The human H^675^Q mutant ASPH has an 80% reduction in enzymatic activity and was subsequently examined to determine if there would be a loss in ability to promote cell proliferation, migration, invasion, and colony formation compared to the “wild type” protein. Figure 5 illustrates the stimulatory effects of WT-ASPH overexpression in PC cells on proliferation, migration, invasion, and colony formation, as well as the activation of Notch responsive genes. However, the mutant ASPH H^675^Q inhibited cell proliferation, migration, invasion, and colony formation as well as reduced HES1, HEY1, CD44, EpCAM, c-Myc, MMP2/9, cyclin D3, and PCNA gene expression in PC cells.

### ASPH generates a malignant phenotype in PC through Notch activation

Notably, ASPH overexpression activated Notch responsive genes such as HES1, HEY1, CD44, EpCAM, c-Myc, MMP2/9, cyclin D3, and PCNA (Fig. 2f). To further link ASPH expression to Notch activated genes, we performed shRNA “knockdown” experiments in AsPC-1 cells as demonstrated in Fig. 3f. Lentiviral induced stable cell lines expressing scrambled shRNA showed the expected high level of endogenous expression of ASPH and prominent upregulation of HES1, HEY1, CD44, EpCAM, c-Myc, MMP2/9, cyclin D3, and PCNA. In contrast, AsPC-1 cells stably transfected with 3 different specific ASPH targeted shRNAs demonstrated downregulation of these Notch responsive genes.

We determined if Notch1 and JAG2 directly interact with and bind to ASPH by co-immunoprecipitation experiments. As displayed in Fig. 4d, e, Notch1 and JAG2 directly interact with ASPH. Interestingly, this physical interaction property was maintained with the mutant H^675^Q ASPH construct, suggesting that the protein-protein binding sites between ASPH and Notch1/JAG2 may be independent from the enzymatic activity since a H^675^Q mutation in the catalytic site has been previously shown to reduce β-hydroxylase to 20% of “wild type” (WT) levels [11]. ASPH expression also promoted nuclear translocation of the C-terminal ICN of Notch1 into the nucleus of MIA PaCa2 cells overexpressing WT-ASPH (Fig. 4f). In contrast, nuclear accumulation of activated Notch1 ICN was inhibited with loss of enzymatic activity caused by H^675^Q mutation in ASPH as displayed in Fig. 4f.

### High throughput screening assay for ASPH β-hydroxylase activity inhibitors

**Fig. 6:**
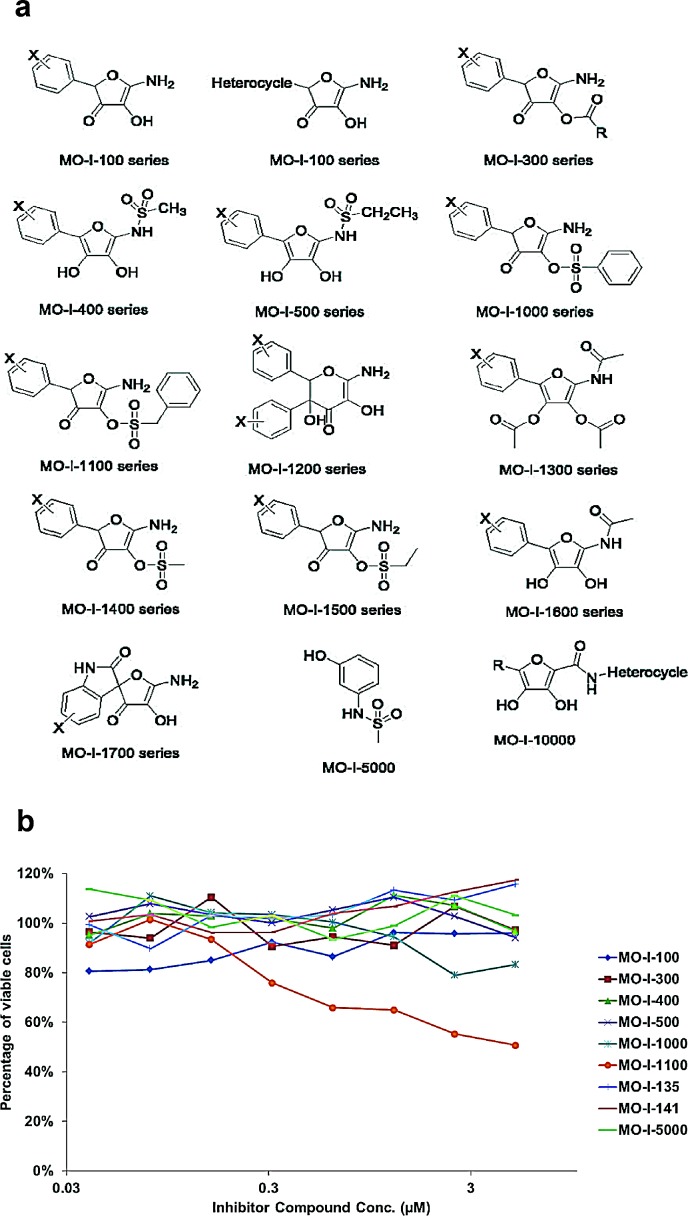
(a) Candidate parent compounds selected and evaluated as potential inhibitors of ASPH β-hydroxylase activity were synthesized on the basis of the crystal structure of the catalytic site in the C-terminal region of ASPH using computer assisted drug design. (b) Effects of SMIs on cell viability of FOCUS HCC cells that highly expressed ASPH on the cell surface as a drug screen for biological activity. Note that MO-I-1100 demonstrated a dose dependent effect on cell viability over a range of 0.03 – 3 μM whereas other compounds such as MO-I-100, 300 and 400 for example, showed little, if any, activity.

An assay to measure ASPH β-hydroxylase activity had been established as described [11, 12]. This high throughput system was used to screen for potential small molecule inhibitors (SMIs) of β-hydroxylase activity that were synthesized as shown in Fig. 6a. When inhibitors of ASPH β-hydroxylase activity were identified, a functional MTT assay over a range of drug concentrations was performed to assess their effects on cell viability and growth. Examples of positive and negative results among several candidate compounds are depicted in Fig. 6b.

These SMIs were rationally designed based upon the aminohydroxyfuranone ring core. There was a “strong hit” with one of the MO-I-1100 compounds since it reduced β-hydroxylase activity by 80% and cell viability over a range of concentrations (Fig. 6b).

**Fig. 7:**
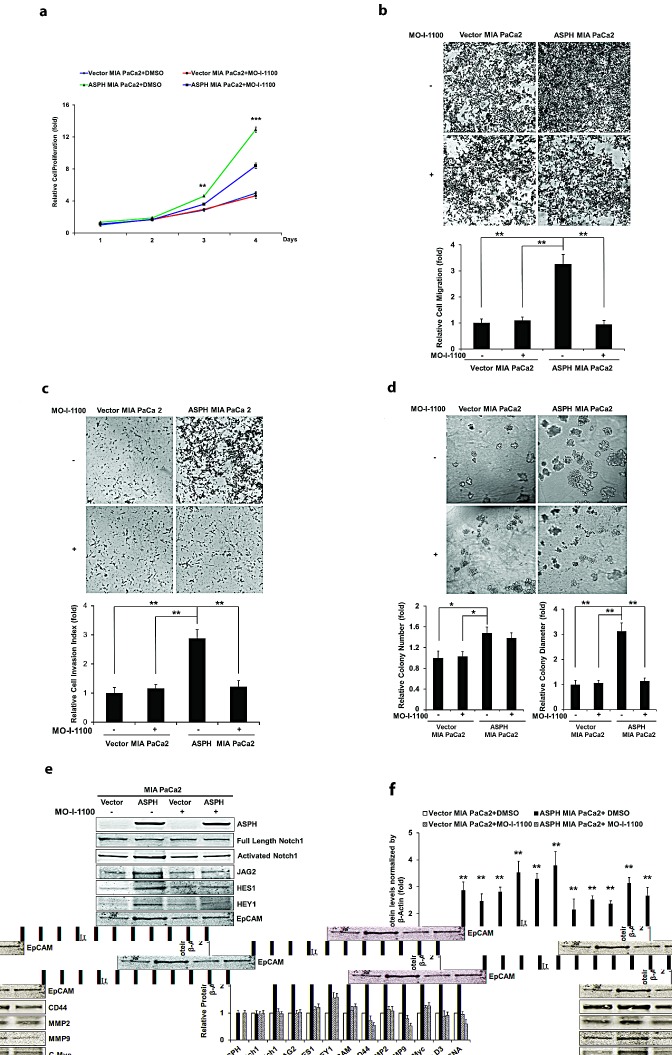
Effect of SMI MO-I-1100 on the PC phenotype induced by exogenous or endogenous high-level of WT-ASPH expression MIA PaCa2 cells stably transfected with empty vector or the “wild type” ASPH construct via lentiviral transfection depicted in Fig. 2. The inhibitory effects of MO-I-1100 on (a) proliferation, (b) migration, (c) invasion, and (d) colony formation of MIA PaCa2 cells were observed. There is a significant reduction in the expression of Notch1 ICN, JAG2, as well as downstream responsive genes HES1, HEY1, EpCAM, CD44, c-Myc, MMP2/9, cyclin D3 and PCNA induced by MO-I-1100 compared to the DMSO treatment (e and f). ^*^p<0.05; ^**^p<0.01; ^***^p<0.001.

The biological effects of reduced ASPH enzymatic activity on generation of a malignant phenotype was evaluated. Figure 7 demonstrates the inhibitory effects of MO-I-1100 on ASPH induced proliferation, migration, invasion and colony formation, as well as Notch signaling activation in MIA PaCa2 cells with high expression of WT-ASPH.

**Fig. 8:**
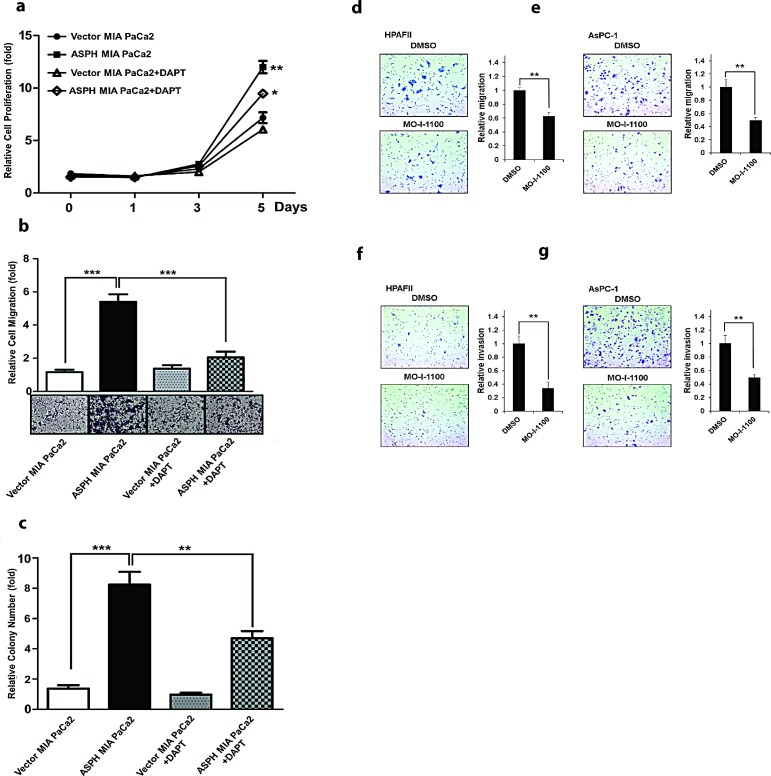
The γ-secretase inhibitor DAPT (at a concentration of 10 μM) reversed the aggressive malignant phenotype exhibited by ASPH overexpression in MIA PaCa2 cells DAPT attenuated ASPH induced (a) cell proliferation, (b) migration and (c) colony formation through inhibition of Notch1 ICN signaling. A SMI (MO-I-1100) exerted inhibitory effects on the PC phenotype induced by endogenous high-level of ASPH expression. A significant reduction in (d, e) migration and (f, g) invasion was observed in HPAFII and AsPC-1 PC cells with high endogenous levels of ASPH (Fig. 1) treated with MO-I-1100 at 5 μM. *p<0.05; **p<0.01; ***p<0.001 when compared with control.

To further link the association between ASPH overexpression and Notch activation, we treated MIA PaCa2 cells with a γ-secretase inhibitor (DAPT) as shown in Fig. 8. Treatment reduced PC proliferation, migration and colony formation. Similar results were obtained with HPAFII and AsPC-1 PC cells which have high endogenous levels of ASPH indicating that enhanced migration and invasion could be inhibited by MO-I-1100, a SMI of ASPH β-hydroxylase activity *in vitro* under these conditions as well (Fig. 8d-g).

### Inhibition of ASPH β-hydroxylase activity reduces PC tumor development and growth in immunodeficient mice

**Fig. 9:**
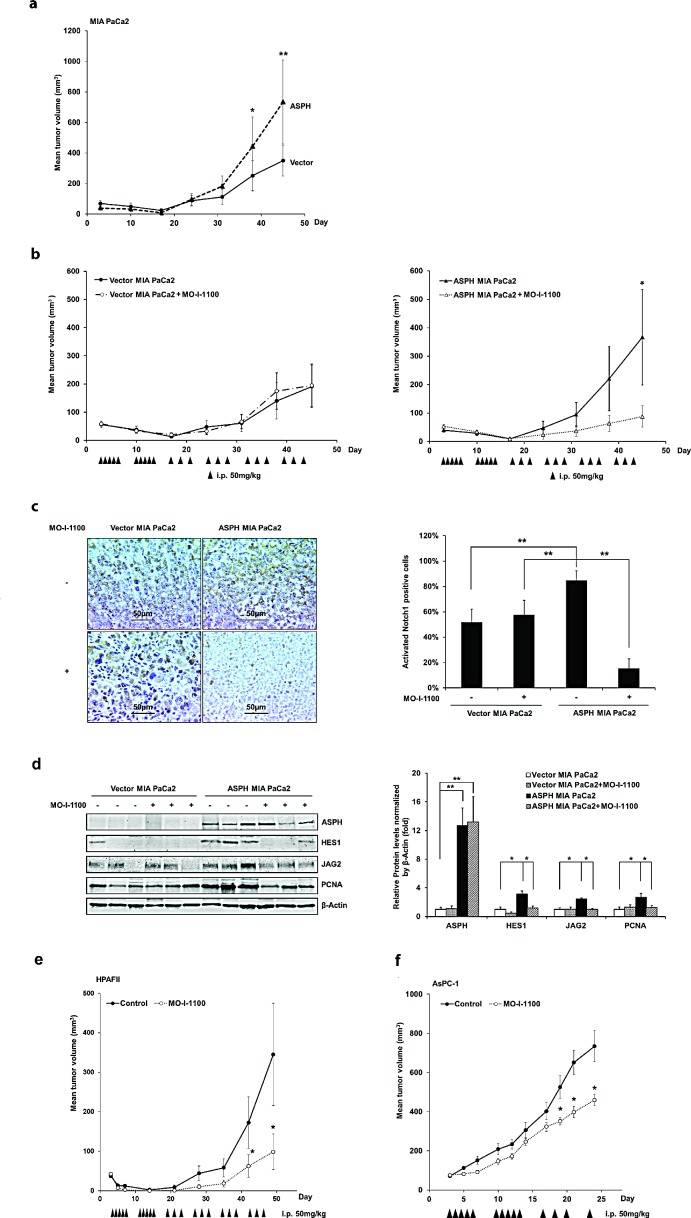
Effect of a SMI (MO-I-1100) on PC tumor growth in subcutaneous (s.c.) tumor model of nude mice (a) ASPH transfected MIA PaCa2 cells had high tumorigenicity compared to MIA PaCa2 cells transfected with empty vector. Growth curve on 8 animals in each group indicated enhanced tumor development induced by ASPH overexpression. (b) represents tumor growth curves derived from MIA PaCa2 cells stably transfected with empty vector or a WT-ASPH expression construct as delivered by lentivirus. Note that anti-tumor effects exhibited by MO-I-1100 were observed only in tumors with exogenous ASPH overexpression. Each group had 10 animals. Effect of MO-I-1100 on Notch signaling *in vivo* has been observed. Tumors taken from the growth curve that were responsive to MO-I-1100 treatment depicted in (b) were analyzed for Notch signaling compared to untreated control. There were 3 tumors taken from the MO-I-1100 treatment group and 3 from the untreated control. (c) demonstrates that the expression of the Notch1 ICN is substantially downregulated by the SMI of APSH β-hydroxylase activity by IHS. (d) represents reduction in the expression of JAG2, as well as Notch activated genes HES1 and PCNA following MO-I-1100 treatment. Similar inhibitory effects of MO-I-1100 on PC tumor growth *in vivo* were observed in (e) HPAFII and (f) AsPC-1 cells induced s.c. tumors. These human PC cell lines had high endogenous expression of ASPH as shown in Fig. 1a. ^*^p < 0.05; ^**^p < 0.01.

Studies were performed to determine if ASPH overexpression can promote tumor growth in Balb/c nude mice inoculated subcutaneously (s.c.) with MIA PaCa2 cells. Tumor growth rates are demonstrated in Fig. 9a, where ASPH overexpression accelerated tumor formation compared to tumors induced by stable vector transfected controls.

It was of interest that MO-I-1100 had no effect on tumor development and growth induced by stably transfected empty vector control MIA PaCa2 (Fig. 9b). Since the only difference between these two cell lines was the presence or absence of exogenous ASPH expression, PC tumor development and growth may be most susceptible to a SMI of β-hydroxylase activity in those tumors that have high levels of ASPH expression.

Furthermore, established large tumors generated by MIA PaCa2 cells stably overexpressing ASPH and vector control were available for analysis of Notch signaling after MO-I-1100 treatment as shown in Fig. 9c, d. Tumor growth that was inhibited by MO-I-1100 treatment was analyzed for Notch1 ICN expression, which demonstrated a reduction in cytoplasmic and nuclear accumulation following treatment as shown in Fig. 9c. In addition, there was a downregulation of Notch responsive genes in these tumors as demonstrated by reduced JAG2, HES1 and PCNA expression as illustrated in Fig. 9d. The antitumor effects of blocking ASPH β-hydroxylase activity was also assessed in immunodeficient mice with two other PC cell lines that have high endogenous ASPH expression (HPAFII and AsPC-1). The results suggest that reducing the β-hydroxylase activity with MO-I-1100 treatment has substantial effects on PC tumor growth as shown in Fig. 9e, f.

## DISCUSSION

The Notch signaling cascade is a highly conserved pathway that principally controls cell fate determination during embryogenesis by facilitating cell-cell communications. It is a major regulator of cell proliferation, migration, and invasion, and plays a prominent role in apoptosis [13]. The transcriptional program mediated by this signaling cascade includes upregulation of the well characterized HES and HEY family of transcription factors. Some of the other well-known and characterized downstream target genes include P21, c-Myc, PDGFR, EGFR, WNT 3/4, PTEN, Bcl-2, cyclin D 1/3, cyclin E1, MMP2/9, CD44, EpCAM and PCNA [14-18]. Several investigations suggest that the expression and activation of Notch receptors and ligands appear to be downregulated in the normal adult pancreas [13], only to re-emerge during pancreatic oncogenesis.

ASPH has negligible to very low expression in normal adult human tissue with the notable exception of the placenta, an invasive tissue, where its expression is robust [19, 20]. ASPH is expressed in many organs during embryogenesis, presumably to promote cell migration for organ development. Its expression is “shut off” in the adult only to re-emerge during oncogenesis where it may be required for generation of malignant phenotypes [11, 12, 21]. Transcriptional regulation of ASPH is provided by tripartite signaling pathways: insulin (IN) and insulin like growth factor 1 (IGF1)/insulin-receptor substrate type 1 (IRS1)/MAPK/ERK, IN/IGF1/IRS1/PI3K/AKT, and WNT/β-Catenin [22, 23]. Post-transcriptional regulation of ASPH is mediated by phosphorylation of GSK3β-related motifs located in the N-terminal region of the molecule [24]. The present study attempts to address several questions central to the function of ASPH in PC. Is ASPH a major activator of Notch signaling in PC to promote cell proliferation, migration, invasion and malignant transformation? Is the enzymatic activity central to its biological function as a transforming protein? Can the β-hydroxylase activity be inhibited by SMIs to produce antitumor effects in preclinical animal models which could be extended to patients in the future? Is the biological activity of ASPH dependent on Notch signaling as a central mechanism for generating its oncogenic properties?.

Activation of Notch signaling may be one of the major factors involved in PC development and growth. There is ample evidence to suggest that the upregulation of Notch receptors and ligands [25] is a common feature of such tumors [26] which leads to enhanced expression of downstream Notch target genes [13, 27, 28]. Notch signaling, which is induced by overexpression of its receptors and ligands, may be required for tumor maintenance [29]. However, characterization of other activators of the Notch cascade in PC and how they may be connected is not well established [13].

ASPH is significantly upregulated in PC patients as shown in Fig. 1. These observations suggest that ASPH may play an important role in PC pathogenesis. We observe that ASPH expression was negative by IHC in grade 1 and 2 PanINs. There is very weak staining for ASPH expression with grade 3 PanINs with more advanced cytological atypia characterized by mitotic figures, pleomorphism and hyperchromatic nuclear staining and prominent nucleoli. Thus, ASPH appears to be overexpressed primarily in the fully transformed malignant phenotype with little, if any, expression in premalignant lesions. These findings suggest that the protein may play an important role in the PC transformation process. Of interest is that metastatic spread of PC to regional lymph nodes was highly positive for ASPH expression (7 lymph nodes were examined derived from 4 different tumors). As shown in Fig. 1d, ASPH staining is correlated with enhanced activated Notch 1 and HES1 expression. It has been reported that ASPH high expression is associated with poor survival of HCC and cholangiocarcinoma patients [21, 30]. We are currently obtaining similar information on PC patients to determine the relationship between ASPH expression in tumor tissue and clinical outcome. ASPH protein may be also involved in other biological processes, besides the Notch pathway, to promote PC development and there is evidence that expression inhibits apoptosis, stimulates cell proliferation and prevents development of senesecence by other non-Notch related cascades (unpublished).

The purpose of measuring the expression profiling of ASPH and Notch (full-length [FL] and intracellular domain [ICN]) pathway components is to identify these PC cell lines with low vs. high endogenous ASPH expression and to subsequently make the stable cell lines with exogenous overexpression of ASPH vs. knockdown of ASPH in the presence of Notch receptors and ligands (for example, JAGs) for further investigations on the molecular mechanisms of PC carcinogenesis. Lentiviral induced ASPH overexpression in MIA PaCa2 PC cells with low endogenous expression levels was notable for translocation of the protein to the tumor cell surface. This ultimately conferred a malignant phenotype, characterized by enhanced proliferation, migration, invasion and colony formation. The β-hydroxylase activity of ASPH appeared central to its function, as the expression of the H^675^Q mutant construct, which had an 80% reduction of enzymatic activity reversed this cellular phenotype (Fig. 5).

The development and characterization of SMIs of β-hydroxylase activity was informative. Based on this high-throughput screening approach and the crystal structure of the ASPH catalytic site, a number of potential candidate SMIs were synthesized. We found that a MO-I-1100 compound was a potent inhibitor of ASPH and reduced enzymatic activity by about 80% compared to the “wild type” protein. In this regard, MO-I-1100 substantially inhibited PC cell migration and invasion in HPAFII and AsPC-1 cell lines, which have high endogenous levels of ASPH. In addition, similar effects were observed in MIA PaCa2 cells with stable overexpression of exogenous ASPH. More importantly, MO-I-1100 treatment of nude mice bearing s.c. grown tumors induced by these three PC cell lines reduced tumor growth and progression. It was of interest that MO-I-1100 was effective on PC tumors that expressed ASPH, but appeared inactive in parenteral PC cells with low ASPH levels. These findings raise the possibility that a high level of ASPH expression in human PC tumors may be a useful biomarker for selection of tumors that could respond to SMIs of β-hydroxylase as a potential therapeutic approach.

Little is known regarding the factors and signaling pathways that activate the Notch cascade in PC. The observation that IN/IGF1/IRS1/RAS/RAF/MAPK/ERK, IN/IGF1/IRS1/PI3K/AKT and WNT/β-catenin pathways upregulate ASPH at the transcriptional level and crosstalk with each other [31-33] links these growth factor signaling cascades that are commonly upregulated in PC [34-45] to Notch activation through ASPH. Overexpression of ASPH activates Notch by promoting cleavage of Notch1 ICN to liberate the C-terminal ICN and subsequently upregulates, at the transcriptional level, a number of downstream Notch responsive genes such as HES1, HEY1, CD44, EpCAM, c-Myc, MMP2/9, cyclin D3, and PCNA. Moreover, a mutation (H^675^Q) in the catalytic site, as well as a SMI of ASPH β-hydroxylase activity, reverses ASPH mediated Notch activation. In addition, treatment of ASPH overexpressing PC cells with a γ-secretase inhibitor (DAPT) reduced a phenotype of enhanced migration, invasion and colony formation which suggests that ASPH effector activity may be mediated through Notch signaling.

**Fig. 10:**
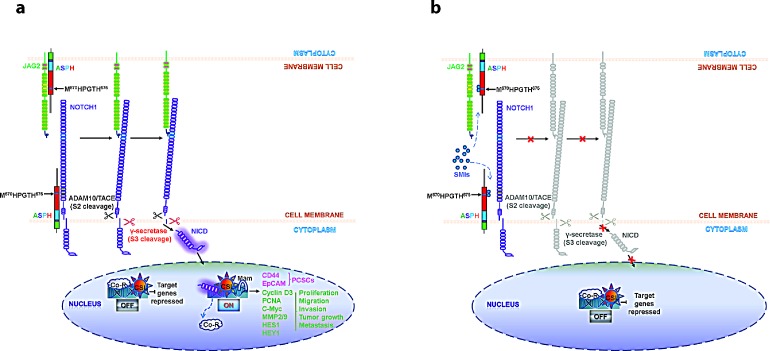
Hypothesis of how ASPH may be involved in the pathogenesis of PC progression through Notch activation [6] The structure of ASPH molecule consists of cytoplasmic (green), transmembrane (purple), luminal region (blue) and catalytic domain (red), which contains the catalytic site (M^670^HPGTH^675^). (a) In the presence of β-hydroxylase activity of ASPH, binding of the JAG ligand (green) on one cell to the Notch receptor (purple) on another cell results in two proteolytic cleavages of the receptor. The ADAM10 or TACE (TNF-α-converting enzyme; ADAM17) metalloprotease (black) catalyzes the S2 cleavage and generates a substrate for S3 cleavage by the γ-secretase complex (red). This proteolytic processing mediates release of the Notch intracellular domain (NICD), which enters the nucleus and interacts with the DNA-binding CSL (CBF1, Su(H) and LAG-1) protein. The co-activator Mastermind (Mam) and other transcription factors are recruited to the CSL complex, whereas co-repressors (Co-R) are released. Thus, the Notch signaling is on and the downstream target genes are expressed: CD44 and EpCAM are markers for cancer stem cells; PCNA, cyclin D3, c-Myc, MMP2/9, HES1 and HEY1 are involved in cell proliferation, migration, invasion, tumor growth and metastasis in PC. (b) SMIs block the catalytic site of ASPH, which leads to loss of β-hydroxylase activity and failure of JAG2 binding to Notch1. Thus, Notch signaling is off and its oncogenic effector is inhibited in PC.

Figure 10 depicts a working hypothesis of how ASPH contributes to PC development and progression. In this scheme, overexpression of ASPH is followed by translocation to the cell surface where it directly interacts with Notch (Fig. 4). Notch receptors contain 36 EGF-like repeats in the extracellular domain (NECD), which are the substrates of ASPH β-hydroxylase. ASPH may facilitate the interactions of the Notch receptors with their ligands (such as JAG and DLL). It is hypothesized that enhanced Notch receptor-ligand interaction leads to the generation of activated Notch1 ICN followed by upregulation of downstream responsive target genes. The net biological effect is to promote cell proliferation, migration, invasion, tumor growth and metastasis, which leads to PC development and progression; this cellular phenotype is attenuated by a SMI of ASPH β-hydroxylase activity.

## MATERIALS AND METHODS

### Cell Culture

The human PC cell lines AsPC-1, BxPC-3, Capan-1, HPAF-II, Hs766T, MIA PaCa2 and Panc-1, as well as hepatocellular carcinoma cell line HepG2 (positive control) were purchased from American Type Culture Collection. All cell lines have been authenticated by short tandem repeat profiling to reduce the frequency of cell misidentification. Cells were cultured at 37°C in a humidified atmosphere containing 5% CO_2_ in corresponding media supplemented with 10% FBS and antibiotics (penicillin and streptomycin). Cells were passaged when they reached 80% confluence.

### Lentiviral infected stable cell line with overexpression or knockdown of ASPH

We established two stable MIA PaCa2 cell lines with overexpression of ASPH, and three stable AsPC-1 cell lines with knockdown of ASPH by specific shRNAs using the lentiviral system (GeneCopoeia, #EX-Z8758-Lv105 and # HSH011500-HIVU6, respectively) as described [7, 8]. Three shRNAs sequences used to target ASPH were as follows: [HSH011500-1-HIVU6 (OS217501)] cttctccatgaaatggtac, [HSH011500-2-HIVU6 (OS217502)] ctggaacctgaagtatctc, and ([HSH011500-3-HIVU6 (OS217503)] cctgaggataatcctgtag.

### Western Blot Analysis

Cell lysates were separated by SDS PAGE and transferred to nitrocellulose membranes. Western blot analysis was performed by using primary antibodies against ASPH; JAG1, JAG2, DLL1, DLL4, Notch1, Notch2, Notch3, Notch4, c-Myc, matrix metalloproteinase (MMP)-2, epithelial cell adhesion molecule (EpCAM), and CD44 (Cell Signaling Technology #2620, #2205, #2588, #2589, #4380, #4530, #5276, #2423, #5605, #13132, #2929, #5640, respectively); activated Notch1, HES1 and HEY1 (Abcam ab8925, ab71559, ab22614, respectively); cyclin D3, MMP9, and proliferating cell nuclear antigen (PCNA) (Santa Cruz, sc-56307, sc-21733, sc-7907, respectively). Protein bands were visualized by IRDye® 680RD Infrared Dye and IRDye® 800CW Infrared Dye and exposed on Odyssey image system (LI-COR).

### MTT

Cell proliferation was analyzed using the MTT assay. Sable PC cells (MIA PaCa2 and AsPC-1) transfected with vector, ASPH or ASPH-shRNA (1.5 × 10^4^ cells per well) were seeded in 24 well plates and cultured for 4 days. Fresh media were changed every 2 days. Cells were incubated with MTT solution (Sigma-Aldrich) in corresponding media (10% v/v) at 37°C for 3 h. Then 0.1 N HCI isopropanol was added. Plates were analyzed daily at a wavelength of 570 and 690 nm, respectively. The background absorbance of multiwall plates measured at 690 nm was subtracted from the measurement at 570 nm.

### Wound healing assay

Stable PC cells (MIA PaCa2 and AsPC-1) transfected with ASPH or vector were seeded in a 6-well plate and cultured to reach 80% confluence. Cells were starved for 24 h in corresponding media containing 1% FCS before a scratch was made using a 200 μl pipette tip. The wounded monolayer was washed with PBS, incubated with 5% FCS, and photographed using a microscope. Cells were incubated for an additional 24 h and then photographs were taken of the wounded area. All data were obtained from at least three independent experiments.

### Migration, Invasion and Colony Formation Assays

Assays to measure PC cell migration, invasion and colony formation were performed as described previously [7, 8].

### Generation of a small molecule inhibitor (SMI) of ASPH enzymatic activity

#### Synthesis and characterization of novel SMI for ASPH

Based on the crystal structure of the ASPH catalytic site, computer generated drug design was performed, leading to the synthesis of a series of parent compounds and derivatives likely to fit into the pocket of the catalytic site and inhibit the β-hydroxylase activity. Figure 6 listed some of the parent compounds synthesized and examined for inhibition of β-hydroxylase activity using a high throughput screening assay. Synthesis of SMIs was accomplished in two steps. The first step was a three-component reaction including an aromatic aldehyde, glyoxal bisulfate addition product, and potassium cyanide to yield an arylhydroxytetronimide. In the second step arylhydroxytetronimide was sulfonylated with phenylmethansulfonyl chloride in dry tetrahydrofuran to yield. Compounds were characterized by ^1^H and ^13^C nuclear magnetic resonance, high resolution mass spectroscopy, high performance liquid chromatography, infra-red spectroscopy, melting point, elemental analysis and binding to ASPH by isothermal titration calorimetry.

### Subcutaneous tumor model

This research was approved by the Institutional Animal Care and Use Committee of Rhode Island Hospital. Athymic BALB/c male nude mice (Charles River laboratory) were housed in laminar flow cabinets under specific pathogen-free conditions and studied at 5-6 weeks of age. Stable MIA PaCa2 cells, in exponential phase of growth were transfected with vector or ASPH lentiviral expression. In addition, AsPC-1 and HPAFII cells were harvested, washed, and resuspended in Ca^2+^/Mg^2^-free HBSS, and adjusted to the desired cell concentration. Cell viability was determined by trypan blue exclusion and single-cell suspensions containing >90% viable cells was employed. In the subcutaneous (s.c.) model, tumor cells (5×10^5^ to 5×10^6^ in 0.2 mL of HBSS) were injected s.c. into the left (vector) and right (ASPH) shoulder of the nude mice, respectively. Growth of tumors was monitored by measuring the tumor size with calipers every 3 days. The tumor volume (mm^3^) was calculated using the formula: length × depth × depth × 0.5236. The mice were sacrificed after 2 weeks. The tumors were harvested, weighed and fixed with 10% neutral buffered formalin. Immunohistochemical staining for nuclear accumulation of Notch1 intracellular domain (ICN) and Western blot analysis of Notch signaling molecules were also performed.

### Drug treatment of animal models

The xenograft model employed s.c. injected PC cell lines with high levels of exogenous or endogenous ASPH expression. They were injected s.c. into the right shoulder at a concentration of 5×10^5^ varying to 5×10^6^ cells depending in part on the cell lines employed. These cell concentrations would produce tumors in approximately 100% of control male nude mice. The left shoulder was implanted with vector-transfected PC cell lines for comparison with the right shoulder implanted with a high ASPH expressing cell line such as AsPC-1. Tumor growth rate was monitored every 3 days. The animals were sacrificed when tumors reached 2.5 cm (per request of the Animal Studies Committee). Tumors were dissected, weighed, frozen and stored to measure Notch signaling expression by immunohistochemistry (IHC) and Western blot analysis. PC cell lines were allowed to form established tumors before initiation of treatment with SMIs so that measurements of the above parameters could be made before and after therapy with MO-I-1100 (a comparison between initially untreated tumors and treated residual PC). Eight to 10 animals were employed in each group (treatment and control). The dose range of MO-I-1100 was 20, 40, and 60 mg/kg administered intraperitoneally (i.p.).

### Immunohistochemistry

Tissue microarrays (TMAs) containing pathologic specimens from 104 individuals with state I/II PDAC were used for IHC. Those patients had undergone pancreaticoduodenectomy at Lifespan Rhode Island and the Miriam Hospitals, Warren Alpert School of Medicine, Brown University. The study was approved by the Institutional Review Board of Lifespan Rhode Island and the Miriam Hospitals. Immunohistochemical staining was conducted on 4-um formalin-fixed paraffin-embedded (FFPE) unstained sections using the FB-50 mAb. The sections were deparaffinized in xylene and rehydrated in a descending ethanol gradient. Antigen retrieval was performed using Citric Acid Based Antigen Unmasking Solution (Vector Laboratories, Burlingame, CA) in a microwave pressure cooker (Nordic Ware, Minneapolis, IN) for 6 min at full power, followed by a 30 min cool down process. Endogenous peroxidase activity was quenched by a 30 min treatment with 3% hydrogen peroxide in methanol. The remaining steps of the staining procedure, including blocking, secondary antibody incubation, and ABC reagent addition, were performed using the VECTASTAIN Elite ABC Kit (Vector Laboratories, Burlingame, CA) according to the manufacturer’s protocol. Primary antibodies were diluted in TBS/0.1% Tween-20 with 5% normal goat serum and were incubated at 4°C overnight. Color development was performed using DAB Tablets (Wako Chemicals, Richmond, VA) as a substrate per manufacturer’s instruction. Finally, the sections were counterstained by hematoxylin, dehydrated in an ascending ethanol gradient, and mounted with VECTSHIELD Mounting Medium (Vector Laboratories, Burlingame, CA).

### Semi-quantitative measurement of ASPH expression levels

IHC stained slides of PC tissue microarrays were observed at 400× magnification and the staining results were scored. Tumor cells were evaluated for ASPH expression with comparison to surrounding normal pancreas, acute/chronic pancreatitis, normal intestine, and pancreatic neuroendocrine tumors.

### ASPH mutant and shRNA constructs

We inhibited ASPH function by generating a mutant H^675^Q construct, which reduces β-hydroxylase activity to 20% of “wild type” (WT) level, or by shRNAs “knockdown”. The structures of WT-ASPH and H^675^Q mutant expression constructs that were inserted into the pCDNA3 vectors are shown in Fig. 4. Inclusion of a c-Myc tag allowed for discrimination between exogenous and endogenous protein levels. Empty vector, WT-ASPH and H^675^Q-ASPH overexpressing stable MIA PaCa2 PC cell lines were established by using the lentiviral expression system (GeneCopoeia). The ASPH H^675^Q mutant, which substantially reduced the β-hydroxylase activity, was compared to the WT-ASPH with respect to effects on cell proliferation, migration, invasion, and colony formation of stable MIA PaCa2 cells. These constructs were also tested for inhibition of Notch signaling as a downstream effector of ASPH biological activity. The ASPH protein level was confirmed by Western blot and immunofluorescent staining. The ASPH overexpressing MIA PaCa2 stable PC cell line is shown in Fig. 1f. Notch signaling components were assessed by measuring expression profiling of ASPH-Notch network genes through Western blot analysis. Expression levels of activated Notch1 and HES1 were evaluated by immunohistochemistry staining in tissue samples derived from PC patients as shown in Fig. 1d.

### High throughput screening assay for β-hydroxylase activity inhibition

The information on the strategy, methods and development of an assay to measure ASPH enzymatic activity is as described [11, 12].

### Statistical analysis

All results were expressed as mean ± SD. Statistical analyses were performed with the two-tailed Student’s t-test or Wilcoxon rank sum test between control and treatment groups using SPSS software. Due to the normal distribution of the results, comparisons between PC tumor and adjacent non-neoplastic tissue were evaluated by paired t-tests. A *p* value of <0.05 was considered statistically significant.
