# Correction: Aspartate β-hydroxylase expression promotes a malignant pancreatic cellular phenotype

**DOI:** 10.18632/oncotarget.27315

**Published:** 2019-11-12

**Authors:** Xiaoqun Dong, Qiushi Lin, Arihiro Aihara, Yu Li, Chiung-Kuei Huang, Waihong Chung, Qi Tang, Xuesong Chen, Rolf Carlson, Christina Nadolny, Gregory Gabriel, Mark Olsen, Jack R. Wands

**Affiliations:** ^1^ Department of Biomedical and Pharmaceutical Sciences, College of Pharmacy, The University of Rhode Island, Kingston, RI, USA; ^2^ Current address: Department of Internal Medicine, College of Medicine, The University of Oklahoma Health Sciences Center, Oklahoma City, OK, USA; ^3^ Liver Research Center, Rhode Island Hospital, Warren Alpert Medical School, Brown University, Providence, RI, USA; ^4^ Department of Chemistry and Biochemistry, Kennesaw State University, Kennesaw, GA, USA; ^5^ Department of Pharmaceutical Sciences, College of Pharmacy–Glendale, Midwestern University, Glendale, Arizona, USA

**This article has been corrected:** Due to errors during processing, Figure 5D contains an incorrect image of H675Q MIA PaCa2. In addition, in Figure 7B, the images of ASPH MIA PaCa2 treated with or without MO-I-1100 were also incorrect. In panel 7D, the image of Vector MIA PaCa2 (upper left) was incorrect as well. The corrected figures 5 and 7 are shown below. The authors declare that these corrections do not change the results or conclusions of this paper.

Original article: Oncotarget. 2015; 6:1231–1248. 1231-1248. https://doi.org/10.18632/oncotarget.2840

**Figure 5:**
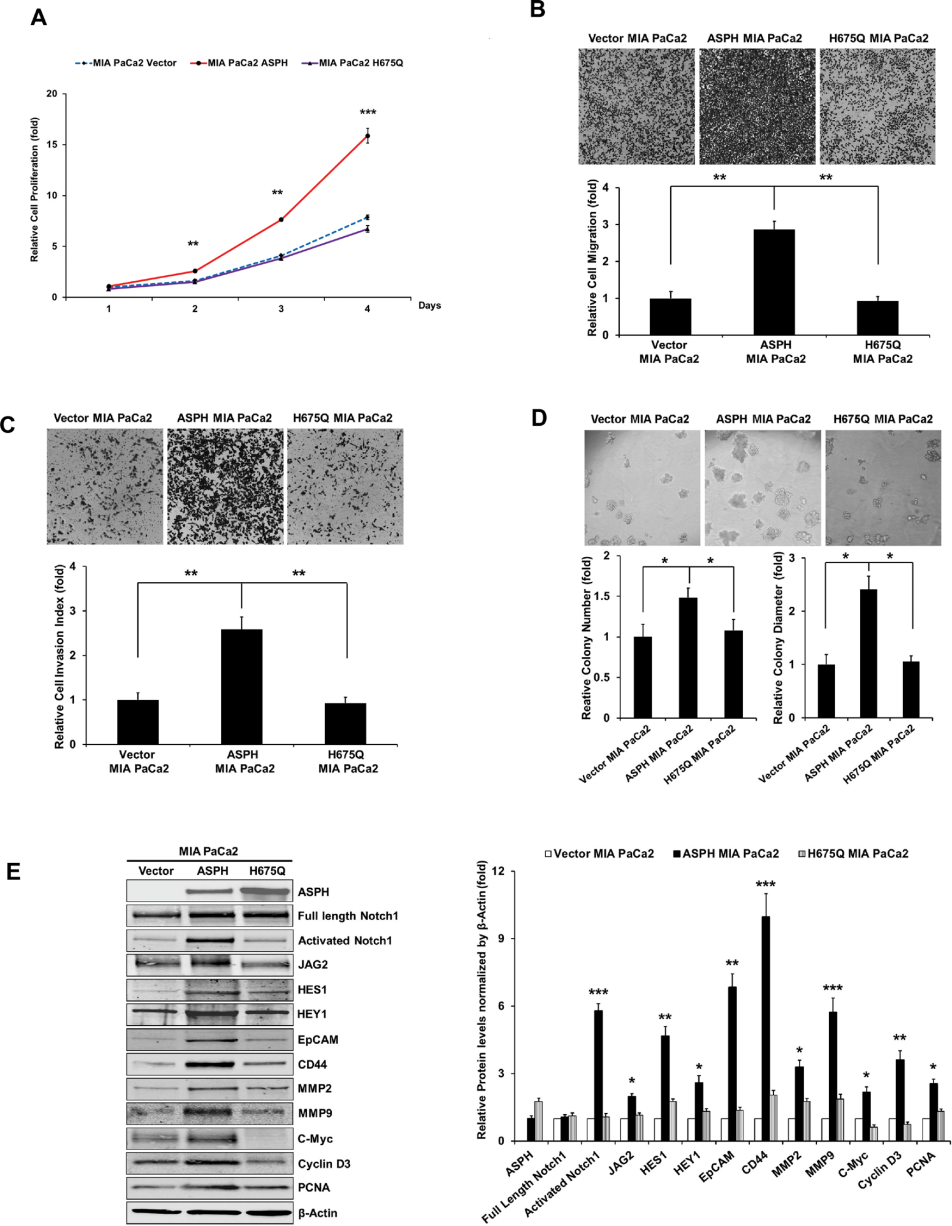
The β-hydroxylase activity of ASPH is required for its transforming activity. MIA PaCa2 cells were stably transfected with empty vector (EV), “wild type” (WT)-ASPH, or H^675^Q-ASPH mutant and the levels of expression were confirmed by Western blot. There is low-level endogenous ASPH in MIA PaCa2 cells as shown here and in Fig. 1. Effects of EV, WT-ASPH and H^675^Q-ASPH on (**A**) cell proliferation, (**B**) migration, (**C**) invasion, and (**D**) colony formation are significantly different. (**E** and **F**) represents a Western blot demonstrating WT-ASPH induced activation of Notch signaling as determined by increased levels of Notch1 ICN, JAG2, as well as increased expression of downstream responsive genes HES1, HEY1, EpCAM, CD44, c-Myc, MMP2/9, cyclin D3 and PCNA. In contrast, the mutant H^675^Q ASPH construct shows significantly reduced activated Notch1 ICN, JAG2, as well as HES1, HEY1, EpCAM, CD44, c-Myc, MMP2/9, cyclin D3 and PCNA. The results suggest that the β-hydroxylase activity of ASPH is essential for Notch signaling activation. ^*^ *p*<0.05; ^**^ *p*<0.01; ^***^ *p*<0.001.

**Figure 7:**
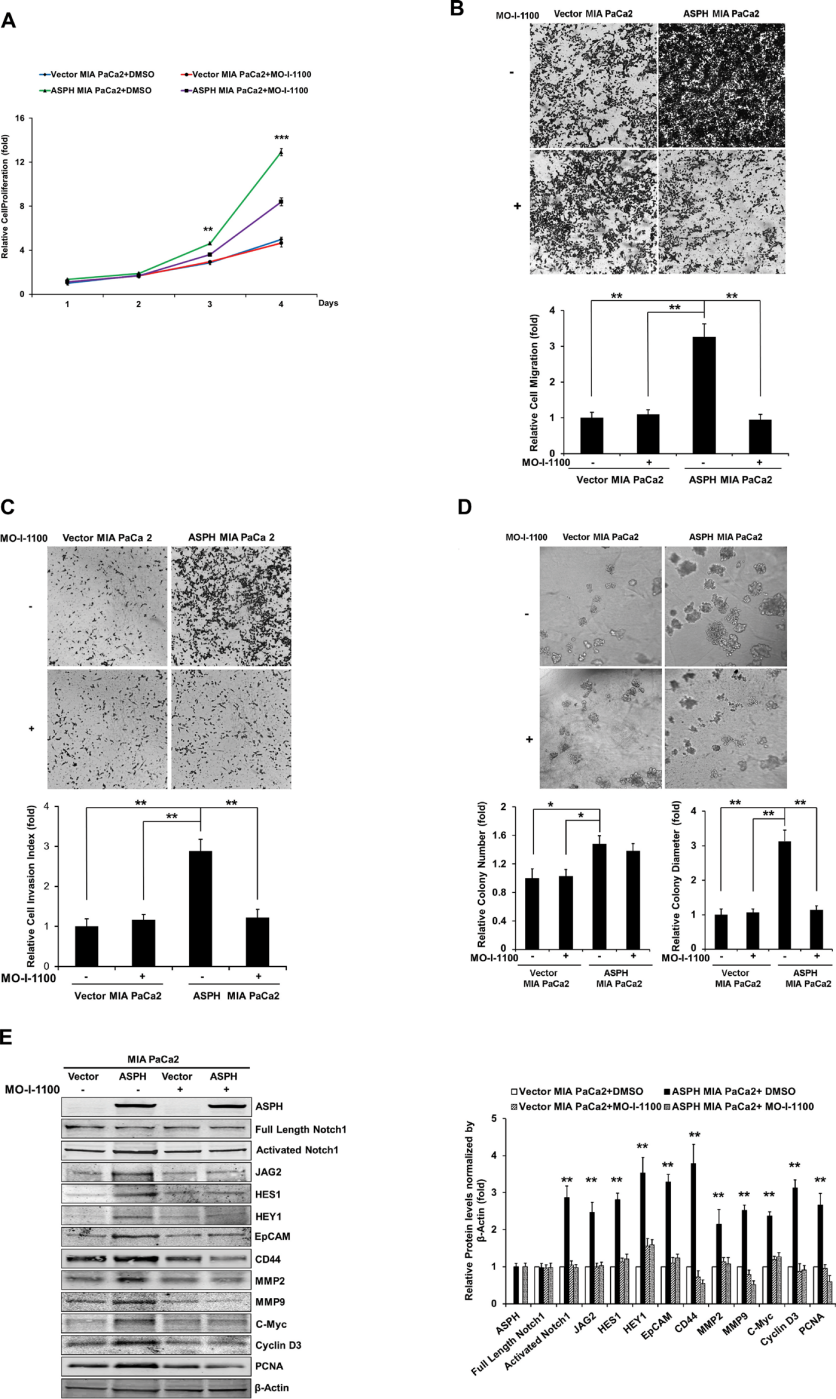
Effect of SMI MO-I-1100 on the PC phenotype induced by exogenous or endogenous high-level of WT-ASPH expression. MIA PaCa2 cells stably transfected with empty vector or the “wild type” ASPH construct via lentiviral transfection depicted in Fig. 2. The inhibitory effects of MO-I-1100 on (**A**) proliferation, (**B**) migration, (**C**) invasion, and (d) colony formation of MIA PaCa2 cells were observed. There is a significant reduction in the expression of Notch1 ICN, JAG2, as well as downstream responsive genes HES1, HEY1, EpCAM, CD44, c-Myc, MMP2/9, cyclin D3 and PCNA induced by MO-I-1100 compared to the DMSO treatment (**E** and **F**). ^*^ *p*<0.05; ^**^ *p*<0.01; ^***^ *p*<0.001.

